# Intraoperative Ultrasound for the Management of Oral Tongue Cancer: a Systematic Review and Meta‐Analysis

**DOI:** 10.1002/oto2.147

**Published:** 2024-06-06

**Authors:** Ryland N. Spence, Vivienne H. Au, Yan Zhao, Allen L. Feng, Amy F. Juliano, Deborah Goss, Mark A. Varvares

**Affiliations:** ^1^ The Warren Alpert Medical School of Brown University Providence Rhode Island USA; ^2^ Department of Otolaryngology–Head and Neck Surgery New York‐Presbyterian Hospital, Columbia University and Weill Cornell Schools of Medicine New York New York USA; ^3^ Department of Otolaryngology–Head and Neck Surgery Massachusetts Eye and Ear Boston Massachusetts USA; ^4^ Department of Radiology Massachusetts Eye and Ear, Harvard Medical School Boston Massachusetts USA; ^5^ Library Services, Massachusetts Eye and Ear Boston Massachusetts USA

**Keywords:** intraoperative ultrasound, margins, oral tongue carcinoma, resection, ultrasound

## Abstract

**Objective:**

To evaluate for correlation between intraoperative ultrasound (IOUS)‐measured tumor thickness (TT) (uTT) and histopathological TT (hTT), and to compare IOUS‐assisted resection with conventional resection in patients with oral tongue cancers.

**Data Sources:**

Ovid MEDLINE (1946‐2023), Embase.com (1947‐2023), and Web of Science (All Databases 1900‐2023).

**Review Methods:**

Inclusion criteria were the use of IOUS for the management of oral tongue cancer. Studies that did not report quantitative data were excluded. Additionally, studies that were not contributory to meta‐analysis, or a narrative analysis of pooled results were excluded. Selection was carried out by 2 reviewers. A total of 2417 studies were initially identified, with 12 ultimately being included in this review, and 7 included in the meta‐analysis. Data were extracted by 2 investigators and were pooled using a random‐effects model.

**Results:**

Our meta‐analysis reveals a pooled correlation coefficient of 0.92 (95% confidence interval: 0.80‐0.96) for studies comparing uTT to hTT. Studies comparing IOUS‐assisted resection to conventional resection found IOUS‐assisted resection yielded wider nearest margins in all studies reporting this outcome.

**Conclusion:**

IOUS reliably measures TT, similarly to that of histopathology measurement. IOUS‐assisted resection, which allows the surgeon to view the deep extent of tumor invasion, may increase closest radial margin distance compared to conventional resection. IOUS‐assisted resection may represent a more reliable approach to achieving clear margins than conventional resection.

Squamous cell carcinoma of the oral tongue commonly affects patients in the United States and abroad.^1^ Stages I and II disease is primarily managed surgically while advanced disease management is more complex, often involving surgery with adjuvant therapy. Adequate resection margins, particularly, the deep margin which is the most frequently positive margin, are critical to achieving local control and maximizing the chance for survival.^2^, ^3^ Nonetheless, among cancers affecting both men and women, oral cavity cancers have the highest positive surgical margin rate among the 10 most common solid cancers.^4^ Institutional factors (case volume and institutional setting) and clinical factors (tumor stage, tumor grade) are also associated with positive surgical margins.^5^ Better intraoperative management of tumor margins could improve the ability to completely extirpate the tumor and decrease the frequency of positive margins, thus improving local control, potentially decreasing the need for adjuvant therapy for Stages I–II disease and impact downstream overall survival.

Frozen section is the preferred intraoperative tool for the assessment of oral tongue cancer resection margins; however, there is a lack of consensus regarding its proper implementation, and its efficacy is questionable with respect to reliably yielding good outcomes for patients.^6^, ^7^, ^8^, ^9^, ^10^ There is no consensus regarding the sampling technique (tumor bed vs specimen), which may contribute to their inability to improve margin control and ultimately improve outcomes for patients. Studies have even suggested there is no survival benefit to using frozen section compared to gross examination alone.^7^, ^11^ Moreover, initially positive margins resected to negative do not yield the same results as an initially negative margin and have poorer local control.^12^, ^13^ This makes obtaining clear margins during the first resection critical.

Ultrasound (US) has largely been used in preoperative evaluation to assess the extent of oral tongue tumors. Its accuracy in measuring tumor thickness (TT) in this context has been previously described and well correlated to surgical pathology.^14^ Use of intraoperative ultrasound (IOUS) to guide resection of oral tongue cancers was first reported in 2001 and presented a promising alternative to traditional resection potentially allowing surgeons to reliably assess margin status during resection, and limiting the number of positive margins.^15^ Since then, there have been numerous studies demonstrating the utility of IOUS for the management of oral tongue cancer. Here, we present a systematic review and meta‐analysis of 12 original articles that report the use of IOUS for the management of oral tongue cancer and discuss their results and implications.

## Methods

### Search Strategy

A systematic review was performed by a medical librarian (D.G.) following the guidelines of the Preferred Reporting Items for Systematic Reviews and Meta‐analyses. A search of published articles was performed via Ovid MEDLINE (1946‐2023), Embase.com (1947‐2023), and Web of Science (All Databases 1900‐2023). Searches were completed on March 31, 2022, and March 30, 2023. The latter search was completed to capture studies that may have been published after the completion of the initial search. Each search utilized an identical combination of controlled vocabulary and keywords focused on IOUS in oral tongue cancer. No filters for language, study design, date of publication, or country of origin were applied. All references were exported into Endnote 7.8 for deduplication and then to Covidence for further deduplication, study screening, selection, and data extraction. Complete details regarding search terms are provided in the appendix (Supplemental Figure S1, available online).

### Study Selection

Two investigators (R.N.S. and V.H.A.) independently reviewed search results and screened the abstracts for inclusion. Inclusion criteria were the use of IOUS for the management of oral tongue cancer. Studies that did not report quantitative data were excluded. Additionally, studies that were not contributory to meta‐analysis or narrative analysis of pooled results were excluded. Studies were considered noncontributory to meta‐analysis or narrative analysis of pooled results if they reported outcomes that could not be directly compared across multiple studies. In our case, we performed meta‐analysis of the correlation between IOUS‐measured TT (uTT) and histopathological (hTT) as this was the most commonly reported data across included studies. Thus, studies that did not report these outcomes were excluded from meta‐analysis. Risk of bias was assessed by 2 independent reviewers (R.N.S. and M.A.V.) using JBI critical appraisal tools for cohort studies and case series. Upon completion of the independent review, the 2 reviewers met to discuss and resolve any differences in the critical appraisal of studies.

### Data Extraction and Analysis

The same 2 investigators extracted data regarding study design, recruitment years, publication year, location of study, as well as tumor measurements (ie, TT, mean nearest margin, etc) and the approach to measurement (ie, IOUS vs histopathology). The most commonly reported measurements that were quantifiable and suitable for meta‐analysis to compare IOUS to other approaches were selected. The only measurement that met these criteria in this study was TT. Studies tended to report Pearson's correlation coefficients comparing uTT to hTT. Thus, we calculated the pooled Pearson's correlation coefficients between uTT and hTT from the included studies using a random‐effects model and assessed the between‐study heterogeneity using the *I* ^2^ statistic. Additionally, to avoid duplication of patients, when studies by the same group had overlapping patients, these patients were removed and Pearson's correlation coefficients were recalculated from raw data. All statistical analyses were performed in R 4.1.3. (R Core Team, 2022). Further, we calculated mean difference between uTT and hTT means when raw data were available to better understand the similarity in measurement between the 2 methods.

## Results

### Search Results

**Figure 1:**
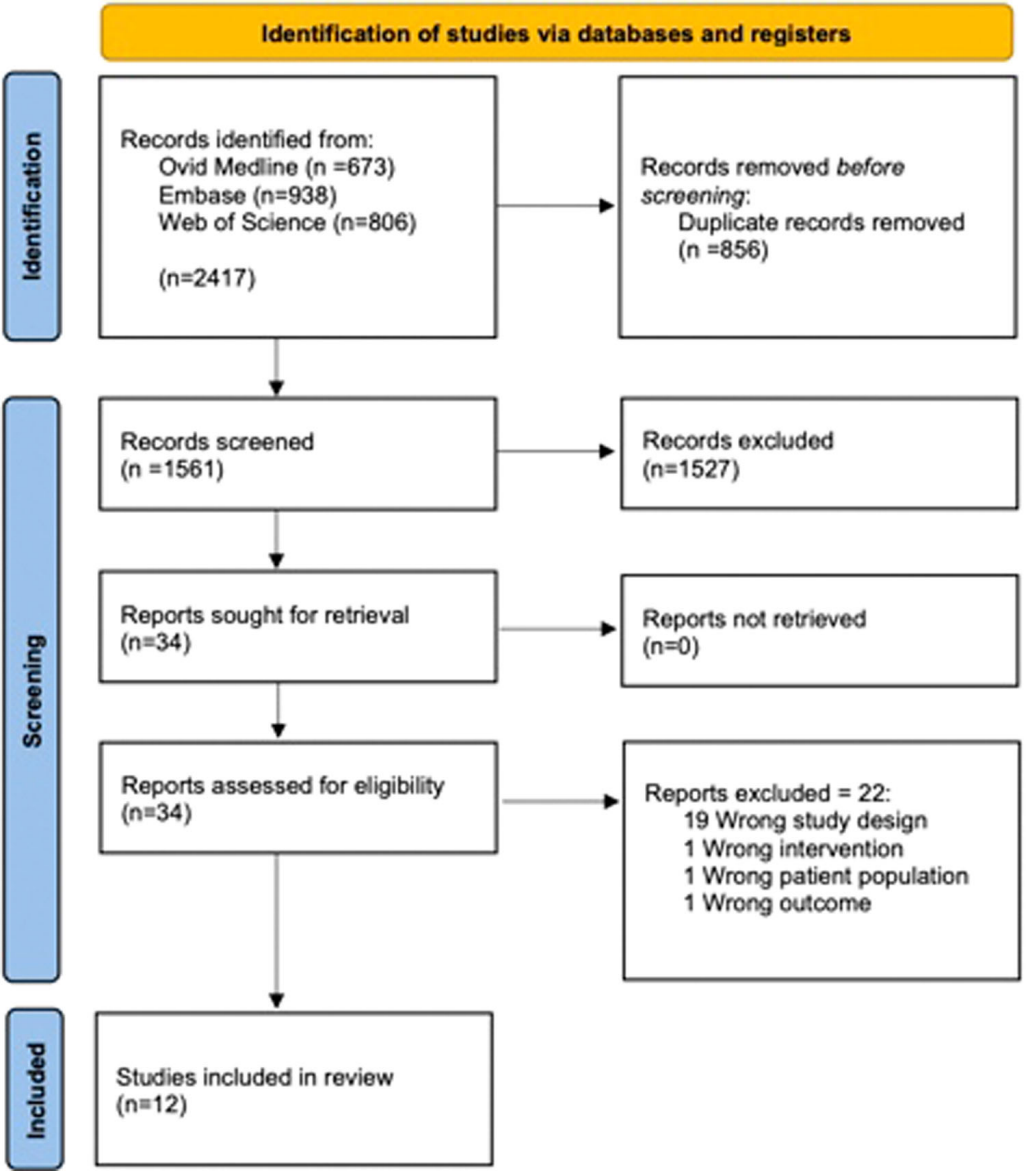
Preferred Reporting Items for Systematic Reviews and Meta‐analyses 2020 flow diagram for new systematic reviews which included searches of databases and registries only.

Our search revealed 2417 articles based on search criteria and after the removal of duplicates, 1561 studies remained. After applying inclusion and exclusion criteria, 12 original articles addressing the use of IOUS for the management of oral tongue cancer were included (Figure 1). Data were reported on an aggregate of 232 IOUS patients. Data from 78 unduplicated patients from 7 studies were used for meta‐analysis. Table 1 summarizes the characteristics of all studies included in both meta‐analysis and narrative review. Tables 2 and 3 list the results of JBI critical appraisal tools for cohort and case series studies for all included studies.^28^, ^29^

**Table 1:** Study Characteristics and Quality of Evidence

References	Mean age, years	IOUS sample	Male (n) female (n)	T stage (n)	Timing of ultrasound	Level of evidence (Oxford)
Songra et al^16^	NR	11	NR	T1 (5) T2 (5) T4 (1)	During resection	4 (prospective case series)
Kodama et al^17^	61.6^a^	13	M (8) F (5)	T1 (4) T2 (9)	Immediately prior to resection	4 (prospective case series)
Tarabichi et al^18^	62^a^	12	M (6) F (6)	T1 (11) T2 (1)	Immediately prior to resection	4 (retrospective case series)
Yoon et al^19^	63^a^	20	M (13) F (7)	NR	Immediately prior, during, and after resection	4 (retrospective case series)
Neha et al^20^	NR	19	NR	T1/T2 (NR/NR)	Immediately prior, during, and after resection	4 (prospective case series)
Yoon et al^21^	59.6	11	M (13) F (6)	NR	Immediately prior, during, and after resection	4 (retrospective case series)
de Koning et al^22^	59.9	10	M (7) F (3)	T1 (3) T2 (9) T3 (1)	Immediately prior and after resection	2b (retrospective cohort)
Bulbul et al^23^,^b^	59.1	23	M (15) F (8)	T1 (13) T2 (8) T3 (2)	Immediately prior, during, and after resection	2b (retrospective cohort)
Baek et al^24^,^b^	57	20	M (10) F (10)	T1 (12) T2 (7) T3 (1)	Immediately prior to resection	2b (retrospective cohort)
Au et al^25^,^b^	63	19	NR	T1 (7) T2 (12)	During resection	4 (retrospective case series)
Nilsson et al^26^,^b^	63	34	M (22) F (12)	T1 (17) T2 (7) T3 (10)	During and after resection	2b (retrospective cohort)
de Koning et al^27^,^b^	58.9	40	M (23) F (17)	T1 (15) T2 (18) T3 (7)	Immediately prior and after resection	2b (retrospective cohort)

Abbreviation: IOUS, intraoperative ultrasound.

^a^ Median age.

^b^ Not included in meta‐analysis.

**Table 2:** JBI Critical Appraisal Tool for Cohort Studies

References	Q1	Q2	Q3	Q4	Q5	Q6	Q7	Q8	Q9	Q10	Q11	Total
Baek et al^24^	Yes	Yes	Yes	Yes	No	Yes	Yes	Yes	Yes	NA	Yes	9/11
Bulbul et al^23^	Yes	Yes	Yes	Yes	No	Yes	Yes	Yes	Yes	NA	Yes	9/11
de Koning et al^22^	Yes	Yes	Yes	Yes	No	Yes	Yes	Yes	Yes	NA	Yes	9/11
de Koning et al^27^	Yes	Yes	Yes	Yes	No	Yes	Yes	Yes	Yes	NA	Yes	9/11
Nilsson et al^26^	Yes	Yes	Yes	Yes	Yes	Yes	Yes	Yes	Yes	NA	Yes	10/11

**Table 3:** JBI Critical Appraisal Tool for Case Series

References	Q1	Q2	Q3	Q4	Q5	Q6	Q7	Q8	Q9	Q10	Total
Kodama et al^17^	Yes	Yes	Yes	No	No	Yes	Yes	Yes	Yes	Yes	8/10
Songra et al^16^	Yes	Yes	Yes	No	No	No	Yes	Yes	No	Yes	6/10
Neha et al^20^	Yes	Yes	Yes	No	No	No	No	Yes	Yes	Yes	6/10
Tarabichi et al^18^	Yes	No	No	No	Yes	Yes	Yes	Yes	Yes	Yes	7/10
Yoon et al^19^	Yes	Yes	Yes	No	No	Yes	Yes	Yes	Yes	Yes	8/10
Yoon et al^21^	Yes	Yes	Yes	No	No	Yes	Yes	Yes	No	Yes	7/10
Au et al^25^	No	Yes	Yes	No	Yes	Yes	Yes	Yes	No	Yes	7/10

### Measurements Reported

TT, defined as the distance from the surface of the tumor to the deepest point of invasion, was the most commonly reported measurement and was found in 7 of the 12 studies.^15^, ^16^, ^17^, ^18^, ^19^, ^22^, ^23^, ^24^, ^30^, ^31^ Five studies used Pearson's correlation test to compare uTT with hTT, and the Pearson's correlation coefficients could be extracted from the remaining 2 studies which reported uTT and hTT data sets. Thus, our meta‐analysis comparing uTT and hTT included 7 studies.

Margin clearance and depth of invasion (DOI) were the second and third most commonly measured values used to compare IOUS against histopathology.^16^, ^18^, ^22^, ^23^, ^24^, ^31^, ^32^ Additionally, 5 studies compared IOUS‐guided resection to conventional resection using various metrics including margin status (free, close, involved), TT, deep margin, DOI, length/width of tumor, among others.^22^, ^23^, ^24^, ^26^, ^27^ However, the heterogeneity in data reporting and statistical analysis of margin clearance, DOI, and other parameters across studies precluded a pooled analysis, and as a result, these parameters were included only in the systematic review.

### IOUS Measurements Correlate With Final Histopathology

**Figure 2:**
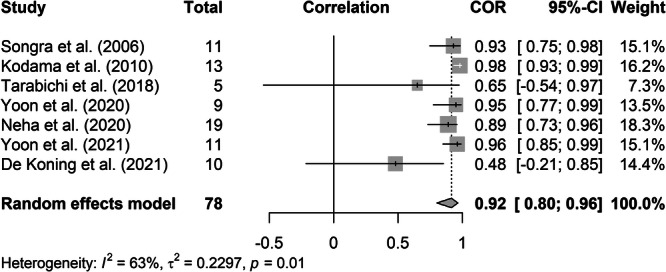
Forest plot showing pooled correlation of studies comparing uTT to hTT. CI, confidence interval.

In our meta‐analysis, Pearson's correlation coefficients between uTT and hTT obtained from the 7 studies were pooled and analyzed to produce a forest plot (Figure 2).^16^, ^17^, ^18^, ^19^, ^20^, ^21^, ^22^ If there was overlap between studies, duplicate patients were removed and Pearson's correlation coefficients were recalculated from raw data. The pooled correlation coefficient of 0.92 (95% confidence interval [CI]: 0.80‐0.96) indicates a strong correlation between uTT and hTT, implying that the US can measure TT similarly to the gold standard of histopathology. Heterogeneity was estimated using the *I* ^*2*^ and yielded a value of 63% (*P* = .01). This indicates moderate heterogeneity between studies. Further heterogeneity assessment was performed with calculation of Cochrane's *Q*, yielding a value of 16.157 (*P* = .013). Influence analysis was then performed by reproducing the meta‐analysis with exclusion of 2 outlier studies, which yielded a pooled correlation coefficient of 0.95 (95% CI: 0.90‐0.97), *I* ^*2*^ 21% (*P* = .28), and Cochrane's *Q* 5.085 (*P* = .279). Additionally, we found the average difference between uTT and hTT means was 0.68 mms, indicating US and histopathology measurements are similar on average.

Two studies made comparisons between IOUS and histopathology regarding the deep margin.^16^, ^19^, ^23^, ^25^, ^32^ Songra et al reported a Pearson correlation coefficient of 0.648 (*P* < .01) between the deep margin measured by IOUS and histopathology.^16^ Baek et al compared the deep margin clearance in IOUS‐assisted resection cases to that of conventional resection, and a student's *t* test revealed a significant difference favoring IOUS‐assisted resection (*P* < .001).^24^

Furthermore, 3 studies compared DOI as measured via IOUS (uDOI) to that of histopathology (hDOI) using correlation analyses.^19^, ^23^, ^25^ Yoon et al, Bulbul et al, and Au et al reported Pearson correlation coefficients of 0.95, 0.9449, and 0.910, respectively, although noting that these studies shared some of the same patients which limits the interpretation of the results.^19^, ^23^, ^25^ This indicates that IOUS may measure DOI similarly to histopathology.

### IOUS‐Assisted Resection compared to Conventional Resection

Five studies compared conventional resection (without IOUS assistance) to IOUS‐assisted resection.^22^, ^23^, ^24^, ^26^, ^27^ These studies reported various metrics; however, most reported the nearest margin status and margin clearance obtained by IOUS‐assisted resection in comparison to the conventional approach.

In 3 studies, IOUS‐assisted resection yielded a wider average nearest margin than conventional resection (*P* < .05). de Koning et al found the mean nearest margin with IOUS to be 4.9 mm, which was significantly greater than the mean of 3.5 mm without US (*P* = .046).^22^ Another study by the same group, which included patients in the previous study, reported similar findings, where IOUS resulted in a mean nearest margin of 4.9 mm compared to 3.4 mm (*P* = .002).^27^ Similarly, Bulbul et al also found a significantly wider mean nearest margin with IOUS (6.3 mm) when compared to without US (4.33 mm; *P* = .018).^23^

Two studies comparing the average deep margin yielded from IOUS‐assisted resection to that from conventional resection reported mixed findings. Baek et al found the mean nearest deep margin with IOUS to be 9.8 mm compared to 4.0 mm without US (*P* ≤ 0.001).^24^ Bulbul et al reported the mean nearest deep margin as 8.5 mm for IOUS‐assisted resection and 6.7 mm for conventional resection, but the difference was not statistically significant (*P* = .18).^23^

In terms of margin clearance, de Koning et al found that IOUS led to more frequently free margins (defined as ≥5 mm), and less frequently positive margins (defined as <1 mm) than conventional resection (55% vs 16%, *P* < .001 and 5% vs 15%, *P* < .001, respectively).^27^ Nilsson et al also found that IOUS led to a decrease in margin positivity compared to conventional resection (2.9% vs 11.8%); however, this result was not statistically significant (*P* = .200).^26^ Finally, Bulbul et al reported a smaller percentage of patients with deep margin <5 mm in the IOUS‐assisted resection cohort (22%) when compared to conventional resection (33%), but the difference was not statistically significant (*P* = .390).^23^

## Discussion

Oral cavity cancers have the third highest positive margin rate among the most common solid cancers. When considering cancers that affect both men and women, oral cavity cancers have the highest rate of positive margins.^4^ This is in part because our most commonly used intraoperative assessment of margin status—frozen section—has unreliable efficacy when it comes to improving positive margin rates.^7^, ^11^ Moreover, studies have shown that re‐resection to a clear margin following a close or positive frozen resection margin does not yield the same results as an initially negative margin when using local recurrence as the primary end point.^12^ This is in large part due to the plasticity of the tongue, the change in position of the tumor bed, and the known difficulty of the surgeon relocating points of concern after the resection has been performed.^33^ There is a need for an approach that enables surgeons to reliably evaluate the 3‐dimensional margins of the tumor intraoperatively, thus yielding a successful first resection and eliminating the need for re‐resection. IOUS is a promising technique pursuant to this goal.

US has been used in the management of oral tongue cancers for decades; however, its use has been primarily aimed at preoperative examination of tumors. Numerous studies have described the close correlation of preoperative uTT with that of hTT, and recent studies have described its reliability in measuring DOI.^34^ Preoperative US has been compared to magnetic resonance imaging (MRI) and computed tomography (CT) in prior studies. Results have been mixed regarding comparisons to MRI.^35^ However, those comparing US to CT tend to reveal US as superior for lesions measuring <5 mm.^34^, ^35^, ^36^, ^37^ Further, US is more cost‐effective compared to MRI for preoperative imaging of lesions confined to the oral tongue.^35^

**Figure 3:**
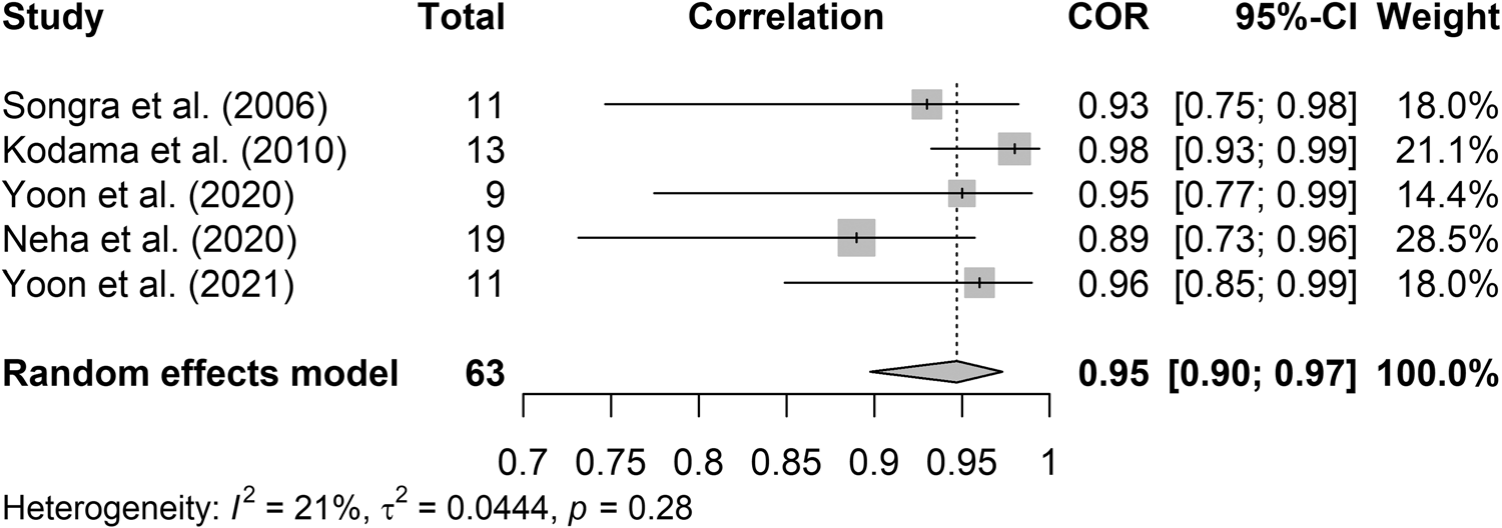
Forest plot showing pooled correlation of studies comparing uTT to hTT, with removal of 2 outlier studies. CI, confidence interval.

The studies included herein focused on the use of IOUS for aiding resection. Studies that compared uTT to hTT found that IOUS was reliable for this measurement, with a pooled correlation of 0.92, indicating a strong correlation between the 2 methods. However, this analysis is limited by moderate heterogeneity, largely due to the small sample sizes of included studies. Further, wide confidence intervals in 2 included studies likely contributed significantly to the overall heterogeneity in the meta‐analysis ^22^, ^38^ This is further supported by the results displayed in Figure 3, showing the reproduction of the meta‐analysis after exclusion of 2 outlier studies. Measures of heterogeneity (*I* ^*2*^ and Cochrane's *Q*) decreased to 21%, and 5.085, respectively, with neither being statistically significant. Moreover, there is overlap among patients from the same groups. This was addressed in the meta‐analysis by removal of duplicate patients using raw data. However, the same was not possible in our narrative analysis of studies, and thus is a limitation.

While the correlation between uTT and hTT has been previously reported in the context of preoperative US, our meta‐analysis focuses entirely on the intraoperative application of US for oral tongue cancer.^14^ The fact that the strong correlation between uTT and hTT persists when measurements are taken intraoperatively is promising and speaks to the accuracy of the modality in providing detailed tissue interfaces in real‐time.

Accurate measurement of DOI has become critical in tumor staging and prognosis as we have learned about its role in occult nodal disease.^39^ When examining those studies that use IOUS to measure DOI, uDOI has a strong correlation with hDOI. These findings suggest that IOUS can reliably measure both tumor depth and thickness. It should be noted, however, that the majority of the data reported in the literature is TT, not DOI. Further, no meta‐analysis could have been performed due to the limited number of studies reporting on uDOI, and variability in the reporting of uDOI. Nonetheless, this data along with the strong pooled correlation of uTT and hTT suggest that IOUS can also be a reliable tool in assessing DOI intraoperatively.

Our analysis also demonstrated 5 studies that compared IOUS‐assisted resection to conventional resection. Three of these studies found a statistically significantly wider nearest margin when using US guidance compared to conventional control.^23^, ^24^, ^26^, ^32^ Notably, 2 of the 5 studies found the nearest deep margin to be wider with IOUS‐assisted resection, although the statistical significance was mixed. Additionally, de Koning et al found that their IOUS‐assisted cohort yielded clear margins (≥5 mm) significantly more frequently than the conventional cohort.^22^ Further, Baek et al found IOUS‐assisted resection yielded fewer recurrences on short‐term follow‐up compared to conventional resection.^24^ These results provide promising support for the use of IOUS to aid in first‐pass clearance of margins in oral tongue cancer resection. As re‐resection following an initially positive margin is not equivalent to an initially negative resection as assessed by local recurrence‐free survival, the use of IOUS may help mitigate both positive margins and risk for local recurrence. Nonetheless, larger studies are needed to strengthen the evidence in support of this approach.

Limitations of our review include the design of included studies (ie, mostly retrospective, nonrandomized, and lack of a conventional cohort in most studies), small sample size of meta‐analysis, as well as a lack of consistency between studies regarding what tumor characteristics were measured which prevented additional meta‐analysis from being performed.

## Conclusion

Support for IOUS‐assisted resection of oral tongue cancers continues to grow and shows promise in the ability to improve overall margin clearance. Our meta‐analysis reveals that US can be used to reliably predict histopathological TT intraoperatively, which differs from previous studies that focused on preoperative US. IOUS‐assisted resection may also provide a larger margin of clearance compared to conventional resection.

Nonetheless, IOUS‐aided‐assisted resection seems to provide reliable measurements of TT and may be superior to conventional resection in avoiding positive margin resections and the attendant risk for local recurrence and mortality. However, larger studies with more robust statistical analyses are needed to further validate the reliability of IOUS‐assisted resection for oral tongue cancers.

## Author Contributions

**Ryland N. Spence**, data acquisition, analysis, and interpretation, draft, revision; **Vivienne H. Au**, data acquisition, analysis, and interpretation, draft, revision; **Yan Zhao**, data analysis and interpretation; **Allen L. Feng**, draft, revision; **Amy F. Juliano**, draft, revision; **Deborah Goss**, data acquisition; **Mark A. Varvares**, concept, design, draft, revision.

## Disclosures

### Competing interests

The authors declare that there is no conflict of interest.

### Funding source

None.

## Supporting information


**Figure S1**. Search terms.
