# Supplementary material for: Intraoperative Ultrasound for the Management of Oral Tongue Cancer: a Systematic Review and Meta‐Analysis
Source: OTO Open. 2024 Jun 6;8(2):e147. doi: 10.1002/oto2.147 (PMC11154832; doi:10.1002/oto2.147)
Supplement: Supplementary file 1 — Figure S1. Search terms. [file OTO2-8-e147-s001.docx]

**Embase**

('mouth carcinoma'/exp OR 'oropharynx cancer'/de OR 'tongue cancer'/exp OR 'oral tongue squamous cell carcinoma'/exp OR 'mouth carcinoma*':ti,ab,kw OR 'mouth neoplasm*':ti,ab,kw OR 'head and neck cancer*':ti,ab,kw OR 'tongue cancer*':ti,ab,kw OR 'cancer of the tongue':ti,ab,kw OR 'tongue carcinoma':ti,ab,kw OR 'lingual carcinoma':ti,ab,kw OR 'tongue squamous cell carcinoma':ti,ab,kw OR 'Oral Tongue':ti,ab,kw OR 'tongue neoplasm*':ti,ab,kw OR 'lingual cancer*':ti,ab,kw) OR ((tumor* OR tumour*) NEAR/5 tongue):ti,ab,kw

109,339

'ultrasound'/exp OR Ultrasonograph*:ti,ab,kw OR sonograph*:ti,ab,kw OR ultrasound:ti,ab,kw OR endosonograph*:ti,ab,kw OR 'ultrasonic radiation':ti,ab,kw OR 'ultrasonic imaging':ti,ab,kw OR sonication:ti,ab,kw OR sonification:ti,ab,kw OR 'ultra sound':ti,ab,kw OR Ultrasonograph*:ti,ab,kw OR Echotomograph*:ti,ab,kw OR Echograph*:ti,ab,kw OR (ultrasonic NEAR/5 energy OR irradiation OR measurement OR sound OR wave* OR imaging OR diagnosis OR diagnoses OR tomography):ti,ab,kw

5,833,940

'intraoperative period'/exp OR intraoperative*:ti,ab,kw OR peroperative*:ti,ab,kw OR 'ultrasound-guided':ti,ab,kw OR 'US-guided':ti,ab,kw OR 'Ultrasound-Assisted':ti,ab,kw OR 'US-Assisted':ti,ab,kw OR 'sonography-assisted':ti,ab,kw OR 'sonography-guided':ti,ab,kw OR 'real-time guidance':ti,ab,kw

447,395

1 & 2 & 3 = 822

**Medline**

(Oropharyngeal Neoplasms/ OR exp Tongue Neoplasms/ OR exp mouth neoplasms/ OR "mouth neoplasm*".ti,ab,kw. OR "mouth carcinoma*".ti,ab,kw. OR "head and neck cancer*".ti,ab,kw. OR "tongue cancer*".ti,ab,kw. OR "cancer* of the tongue".ti,ab,kw. OR "tongue carcinoma".ti,ab,kw. OR "lingual carcinoma".ti,ab,kw. OR "tongue squamous cell carcinoma".ti,ab,kw. OR "Oral Tongue".ti,ab,kw. OR "tongue neoplasm*".ti,ab,kw. OR "lingual cancer*".ti,ab,kw.) OR ((tumor* OR tumour*) ADJ5 tongue).ti,ab,kw.

108,925

Exp Ultrasonography/ OR Ultrasonograph*.ti,ab,kw. OR sonograph*.ti,ab,kw. OR ultrasound.ti,ab,kw. OR ultrasonic radiation.ti,ab,kw. OR 'ultrasonic imaging'.ti,ab,kw. OR sonication.ti,ab,kw. OR sonification.ti,ab,kw. OR 'ultra sound'.ti,ab,kw. OR 'Ultrasonographic Imaging*'.ti,ab,kw. OR Echotomograph*.ti,ab,kw. OR Echograph*.ti,ab,kw. OR (ultrasonic ADJ5 energy OR irradiation OR measurement OR sound OR wave* OR imaging OR diagnosis OR diagnoses OR tomography).ti,ab,kw.

4,218,352

Exp "Intraoperative Period"/ OR intraoperative*.ti,ab,kw. OR peroperative*.ti,ab,kw. OR "ultrasound-guided".ti,ab,kw. OR "US-guided".ti,ab,kw. OR "Ultrasound-Assisted".ti,ab,kw. OR "US-Assisted".ti,ab,kw. OR "sonography-assisted".ti,ab,kw. OR "sonography-guided".ti,ab,kw. OR "real-time guidance".ti,ab,kw.

211,375

1 & 2 & 3 = 627

**Web of Science (All databases)**

"Oropharyngeal Neoplasm*" OR "tongue Neoplasm*" OR "mouth neoplasm*" OR "mouth carcinoma*" OR "head and neck cancer" OR "tongue cancer*" OR "cancer* of the tongue" OR "tongue carcinoma" OR "lingual carcinoma" OR "tongue squamous cell carcinoma" OR "Oral Tongue" OR "tongue neoplasm*" OR "lingual cancer*" OR ((tumor* OR tumour*) NEAR/5 tongue)

121,430

Ultrasonograph* OR sonograph* OR "ultrasonic radiation" OR ultrasound OR "ultrasonic imaging" OR sonication OR sonification OR "ultra sound" OR "Ultrasonographic Imaging" OR Echotomograph* OR Echograph* OR (ultrasonic NEAR/5 energy OR irradiation OR measurement OR sound OR wave* OR imaging OR diagnosis OR diagnoses OR tomography)

26,570,010

intraoperative* OR peroperative* OR "ultrasound-guided" OR "US-guided" OR "Ultrasound-Assisted" OR "US-Assisted" OR "sonography-assisted" OR "sonography-guided" OR "real-time guidance"

311,431

1 & 2 & 3 = 734
